# A prospective study on the feasibility of cochlear implantation during the coronavirus disease 2019 crisis and trends of assessment: experience in a UK centre

**DOI:** 10.1017/S0022215121000190

**Published:** 2021-01-13

**Authors:** H Mohammed, L Kennedy, D Whitehead, N Ahmad, A Banerjee

**Affiliations:** Department of ENT, James Cook University Hospital, Middlesbrough, UK

**Keywords:** COVID-19, Cochlear Implants, Prelingual Deafness, Children, Surgery

## Abstract

**Objectives:**

To demonstrate the feasibility of continuing cochlear implantation during the coronavirus disease 2019 crisis and to report on trends of referrals via the neonatal hearing screening programme.

**Methods:**

A prospective case series was conducted on children who underwent cochlear implantation during the coronavirus disease 2019 crisis in the UK and a sample of referrals via the neonatal hearing screening programme. A step-by-step description of peri-operative management is included.

**Results:**

Regionally, between February and May 2020, 106 babies were referred via the neonatal hearing screening programme to paediatric audiology. Eleven children were operated on during the coronavirus disease 2019 study period. None of the 11 children developed coronavirus symptoms.

**Discussion:**

It is widely recognised that the demands of managing the current pandemic may compromise screening, clinical assessment and elective surgery. Time-sensitive issues such as cancer management have gained prominence, but a similar need exists for timely paediatric cochlear implantation.

**Conclusion:**

Implantation in the paediatric population during the coronavirus disease 2019 pandemic is feasible with careful planning.

## Introduction

Coronavirus disease 2019 (Covid-19) was declared a pandemic by the World Health Organization on 11th March 2020.^1^ The Johns Hopkins Institute in the USA keeps a daily record of new and recovered cases, in addition to mortalities, in all countries. Their data show considerable variations from one country to another; at the time of writing this article, countries like Italy and France have flatter curves than other countries such as Brazil and India.^2^ There is concern that Covid-19 may have resurgences or additional peaks as countries ease lockdown measures.^3^

The current Covid-19 pandemic could be seen as a mass casualty incident where the immediate population's needs exceed the resources at hand, and in which effective resource management plays a crucial role.^4^ As part of crisis management, routine patient care, including elective surgery, has largely been cancelled, in order to free up and reallocate resources.^5^ The response to Covid-19 in the UK included reassigning operating theatres and recovery rooms to critical care, and the cancellation or postponement of elective operations.^6^ The National Health Service (NHS) also encouraged remote consultation (via telephone or video) in secondary care as part of its response to Covid-19.^7^ Moreover, NHS England suspended all routine operations for a minimum period of three months, starting from 15th April 2020.^8^

As otolaryngologists were considered to be at high risk of contracting Covid-19 infection,^9^ adjustments to practice were made nationally and internationally. A published report from Hong Kong outlined changes to protect patients that involve screening patients and using personal protective equipment (PPE) to limit cross infection.^10^ A Belgian publication suggested radical changes, such as the use of chisels and cutters instead of drills to drain mastoid abscesses. It had also been suggested that cochlear implantation in prelingual children could be delayed by up to 12 weeks.^9^ There has been significant variation internationally regarding recommendations; for instance, the American Academy of Otolaryngology–Head and Neck Surgery journal *Otolaryngology–Head and Neck Surgery* recommended delaying paediatric cochlear implantation for three to six months.^11^

The majority of ENT surgical procedures within the UK were placed into a ‘defer’ category following guidance by the four Royal Colleges of Surgeons and the NHS,^12^ produced at the start of the Covid-19 pandemic in the UK. However, this guidance also recommended that cochlear implantation in prelingual children with profound hearing loss take place within three months so as not to impact long-term language development.^12^

Such guidance fits with the views of both the British Cochlear Implant Group and the Action on Hearing Loss charity. On 18th March 2020, Professor Helen Cullington, the Chair of the British Cochlear Implant Group, sent an open letter to NHS hospitals asking for cochlear implantation in babies and young children to be treated as a neurolinguistic emergency.^13^ In the absence of clarifying guidelines from the British Cochlear Implant Group at the start of the Covid-19 pandemic, there is anecdotal evidence that some cochlear implant programmes in the UK have struggled to secure support from hospital administrators to offer such services.

Other factors have also contributed to delays in implanting paediatric patients. The redeployment of audiologists^14^ probably led to a reduction in the number of children seen with congenital hearing loss, and a subsequent reduction in referrals to cochlear implant centres. Furthermore, there have been reductions in referrals to secondary care during the Covid-19 crisis, which may include children with congenital hearing loss.^15^ The fall in referrals could be related to patients avoiding healthcare providers entirely or concerns associated with burdening providers with further work during the pandemic.

Early implantation in prelingual patients, who have yet to develop speech and language, was found to have significant effects on performance in standard tests assessing speech, language, functional and social outcomes. For example, a delay in age at implant device switch-on from 9.8 months to 23.5 months was associated (as an independent factor) with a significant degradation in performance in these areas.^16^ It was reported that children implanted at younger than 12 months of age had a smaller deficit in terms of language delay, compared with their normal hearing peers. The older the child at implantation, the larger the deficit.^17^ The ‘critical period’ concept for language learning (in which native language development occurs between 7 and 10 months of age) could explain the benefits of early implantation.^18^ Delays beyond seven and a half months could lead to delayed language ability.^17^ In a more recent study, implanting children up to the age of 11 months resulted in performance within the normal range. Children implanted after this period performed worse than the normal range.^19^

In order to facilitate the timely diagnosis and treatment of children with hearing loss, a newborn hearing screening programme was introduced in England. Babies who fail two automated otoacoustic emission tests and an automated auditory brainstem response (ABR) are referred for further management.^20^ The neonatal hearing screening programme includes the Key Performance Indicator 2 (‘KPI2’) to ensure timely assessment for referrals, which requires that audiological follow up take place within 28 days after screening.^21^

The risk of acquiring Covid-19 within the peri-operative period needs to be balanced against the potential long-term consequences of failing to develop linguistic skills within this time-critical developmental pathway. While the mortality rate for Covid-19 has been reported as approximately 4 per cent,^22^ there have been reports of post-operative mortality as high as 50 per cent in patients who develop Covid-19 symptoms peri-operatively.^23^ Another small but significant study from China reported a 20.6 per cent mortality rate in elective surgical patients who did develop Covid-19.^24^ However, further clarification with the study authors showed that the actual percentage of Covid-19-related mortality in all operated patients stands at 0.046 per cent.^25^ It is also important to highlight the NHS-quoted figure of a 5–7 per cent probability of developing Covid-19 disease while in hospital.^26^

It is anticipated that the ongoing global research collaborative ‘COVIDSurg’^27^ will help in identifying this risk in more detail, although the risk applicable to cochlear implantation may warrant more detailed study within the cochlear implant community.

Considering the significant potential for long-term permanent adverse outcomes of delayed implantation, combined with a lack of clarity regarding when or how restrictions were to be lifted, a consensus decision was reached in our centre so as to offer implantation to prelingual deaf children on our waiting list.

We present our experience of trends and changes to cochlear implantation services and the feasibility of cochlear implant surgery in a paediatric population during the Covid-19 crisis.

## Materials and methods

### Ethical approval

This study received approval from the local audit governance committee. Patient information was stored securely with anonymous evaluation of results.

### Study design and setting

A prospective cohort study was conducted within a regional cochlear implant centre in the UK.

### Data

Collected data included the following. First, it included a sample of referrals via regional neonatal hearing screening programme screeners in the area covered by County Durham, Tees Valley, Hambleton and Richmondshire. As the neonatal hearing screening programme key performance indicators, used by the NHS to ensure timely assessment of neonatal hearing screening programme referrals, require an audiological follow up to take place up to 28 days after screening completion, the sample group was all babies born between 20th February and 20th May in 2018, 2019 and 2020. Second, the data included a sample of national neonatal hearing screening programme referrals for the year 2017–18. Third, the data included implanted patients’ demographics, aetiology of hearing loss, date of referral to the cochlear implant programme, date of surgery, surgical outcome and Covid-19 symptoms after surgery.

### Patients

All paediatric patients implanted between 16th March 2020 and 19th May 2020 were included in this study. Routine care was cancelled within our hospital from 16th March 2020.

### Outcomes

These included: (1) trends of referrals to audiology, as per neonatal hearing screening programme guidance, to our centre and nationally; (2) the time interval between the referral date and surgery; (3) the time interval between the decision to schedule surgery and the actual surgery; (4) surgical outcomes; and (5) the development of Covid-19 symptoms following surgery.

### Procedure

Our centre receives referrals from the entire North East of England region. Each referral is assessed by the cochlear implant team and, if accepted, a key worker, audiologist and implant surgeon are assigned to the child.

Referrals are then triaged into two groups. The first group is the ‘early age’ referrals, which include children referred by local audiology departments. This normally takes place within the first month of the baby's life. Our departmental protocol dictates that this group requires implantation from seven to eight months of age.

The second group, or ‘non-early age’ referrals, is more heterogeneous; it includes children with progressive hearing loss, or with certain types of central pathway deafness such as auditory neuropathy spectrum disorder. In this group, our department follows national guidelines in terms of 18-week targets from referral to treatment.

The primary assessment is undertaken by the team, during which the patient’s history is taken, the diagnostic tests are performed, and the findings are shared with the parents. The child is discussed at the cochlear implant multidisciplinary team meeting (MDT). At the cochlear implant MDT, a decision is made regarding candidacy for implantation with due consideration of medical priorities. The cochlear implant MDT outcome is then discussed with the parents remotely.

A variation in the length of assessments conducted to address inclusion criteria was observed. This is due to investigations such as ABR with the insertion of grommets, and magnetic resonance imaging. Some of these assessments were conducted within the referring centre; repeating them was often not necessary, but was occasionally needed.

If the cochlear implant MDT concludes that surgery cannot be delayed (i.e. in a prelingual child), the name of the child is forwarded to a generic urgent operating theatre list, introduced in our centre towards the end of March (populated by all surgical specialties in hospital). Only procedures that are deemed time-sensitive (i.e. those for which delay could lead to detrimental effects) are forwarded. These procedures fall under ‘category three’ in priority, according to the NHS and the Royal College of Surgeons guidance.^6^

The hospital operating theatre prioritising team meets three times a week and populates operating theatre lists for up to two weeks in advance. Surgical teams are then contacted with dates for their patients. Once dates for our paediatric cochlear implant candidates are received, a cochlear implant surgeon conducts a video consultation with parents. In this consultation, an extended Covid-19 consent^15^ is discussed, with the surgery and subsequent steps explained. The patient is investigated with a Covid-19 swab and chest X-ray 48 hours before surgery. The next day, parents are contacted via telephone for pre-assessment, and they complete a Covid-19 symptom questionnaire.

On the day of surgery, the child is asked to arrive with only one parent. Swab results are checked, with both the child and family members reassessed for symptoms of Covid-19. The child is then reviewed by the anaesthetist and surgeon. The consent form is signed, and the patient is taken to the operating theatre (without the parent, in order to reduce the footfall in operating theatres).

The operating theatres in our hospital have been divided physically into two parts: one for patients who are Covid-19 positive or whose status is unknown, and one for Covid-19 (swab) negative patients.

The child is received by the intubating team, who wear full PPE for the entire operating theatre session in accordance with our trust guidelines.

The child is intubated and proceeds to the operating theatre. All operating theatre staff are also in full PPE. The minimum number of individuals remain in the room during surgery. At the end of the procedure, the child is extubated and taken to an anaesthetic recovery room, where the staff also wear full PPE. The anaesthetic recovery area is located in the operating theatre complex, as per crisis planning team recommendations, to allow for a better turnover of operating theatre cases. Once the child leaves the operating theatre, trust guidelines on equipment decontamination and the 30-minute interval between cases are followed.

On return to the ward, the child is discharged when fit. A remote post-operative review via video link is arranged at one week. Two weeks later, a face-to-face consultation is arranged, during which the ears and post-aural wounds are re-examined, and the cochlear implant is switched on. Both parents are invited for the cochlear implant switch-on, and one audiologist (instead of two) conducts the session. Social distancing is respected. Parents are encouraged to play an active role in the session by distracting the child while the processor is applied and the tests performed. Face-to-face appointments continue to be arranged until an optimal mapping is achieved.

Subsequent medical appointments are kept to video or telephone contact, unless it is essential to see the child face to face. Our implants can be interrogated remotely by our audiology team.

## Results

For the year 2017–2018, 1–5 per cent of screened newborns in England (a total of 139 000) were referred to the audiology department; 534 children were diagnosed with bilateral permanent childhood hearing impairment, including those with auditory neuropathy spectrum disorder.^28^

Regionally, between February and May 2018, a total cohort of 3202 babies were screened in County Durham, Tees Valley, Hambleton and Richmond. Of those, there were 124 referrals to audiology, and 5 children were diagnosed with permanent childhood hearing impairment.

Between February and May 2019, the total cohort was 3094, with 105 referrals and 5 diagnoses of permanent childhood hearing impairment. In 2020, the total cohort for the same period was 3075, with 106 referrals and only 1 child with permanent childhood hearing impairment.

The cohort of patients in the sample period for the years 2018 and 2019 is broadly similar. However, the number of children who met the Key Performance Indicator 2 criteria in 2020 was 47 per cent, which is lower compared to the previous two years (Table 1).

**Table 1.** Children referred from neonatal hearing screening programme regionally to audiology, and referral timing over last three years

Cohort year	Children who met KPI2 (*n* (%))	Children (*n*) who had time delay of their audiological follow up from referral (days)				Appointment pending (*n*)	Deferred audiology assessment (*n*)
		7 days	8–14 days	15–21 days	>21 days		
2018	103 (83)	5	5	2	3		
2019	83 (79)	3	3	3	6		
2020	50 (47)	11	8	7	8	9	2

‘KPI2’ refers to the key performance indicator set by the National Health Service to indicate that children referred need to be seen within 28 days. KPI = key performance indicator

The rate of failure of children to attend their cochlear implant assessment from April 2019 to 31st March 2020 was 31.5 per cent. However, the failure to attend rate for assessments during the Covid-19 pandemic study period (i.e. March to May 2020) was 0 per cent.

**Fig. 1.**
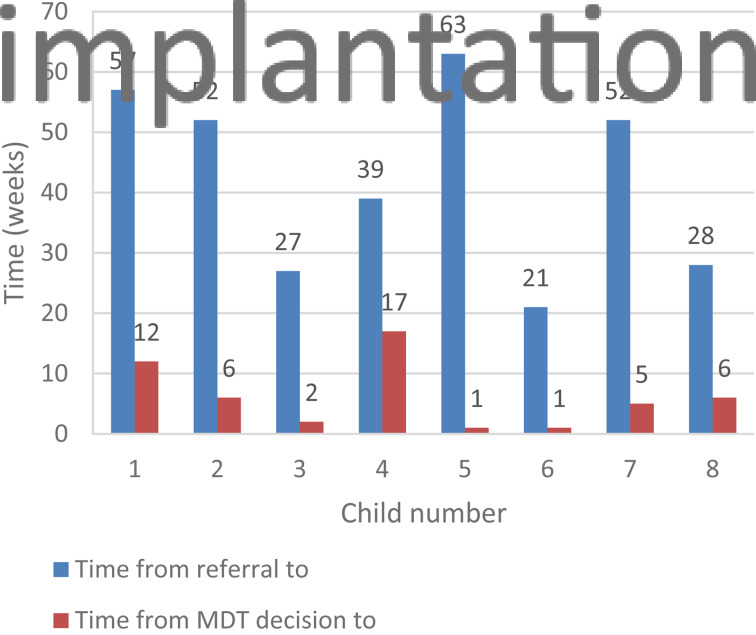
Time from referral to implantation, and time from multidisciplinary team's (MDT's) decision to implantation, for paediatric patients referred in 2018–19. (Child number 8 waited 28 weeks from referral to implantation.)

**Fig. 2.**
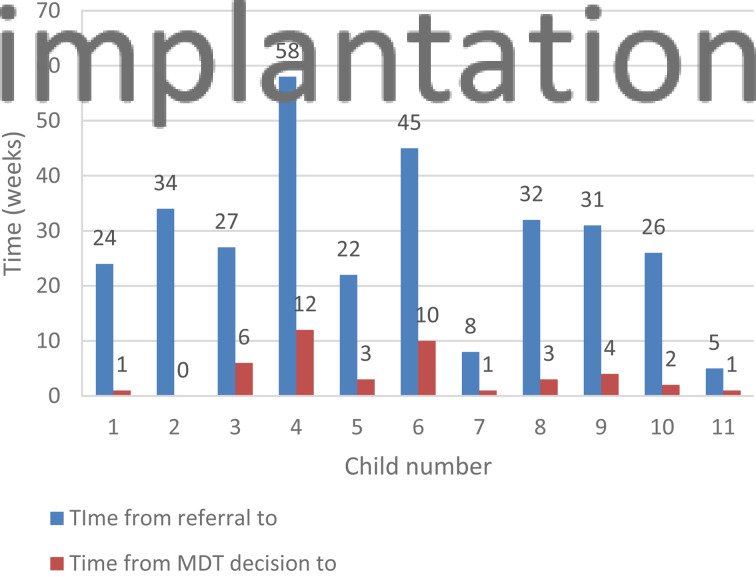
Time from referral to implantation, and time from multidisciplinary team's (MDT's) decision to implantation, for paediatric patients operated on during the study period.

The average time from the cochlear implant MDT's decision to operate to the time of surgery, in the year 2018–19, was 6.25 weeks. This average dropped to 3.9 weeks for children operated on during the Covid-19 study period (Figures 1 and 2).

There were 13 operations in 11 patients within the timeframe of the study. Table 2 shows the study participants’ characteristics.

**Table 2.** Patients’ demographics

Child number	Age at implantation (months)	Sex	Cause of hearing loss	Other co-morbidities	Reasons for urgency	Type of implant
1	8	Female	None found, but strong family history	None	Prelingual	Cochlear CI612
2	9	Male	None found	None	Prelingual	Cochlear CI612
3	37	Female	Connexin 26	None	Prelingual	Cochlear CI612
4	23	Female	Unknown, but suspected undiagnosed syndromic*		Prelingual	Cochlear CI612
5	22	Female	Prematurity of 29 weeks	None	Prelingual, ANSD	Cochlear CI612
6	15	Female	Hereditary, both parents	None	Prelingual	Cochlear CI612
7	23	Female	None found	Single kidney	Prelingual	Advanced Bionics AB Slim J 3D
8	10	Male	Connexin 26; NICU for 5 days	None	Prelingual	Cochlear CI612
9	19	Male	Multifactorial^†^	None	Prelingual, ANSD	Cochlear CI612
10	33	Male	Unknown	Long-sighted	Prelingual	Cochlear CI612
11	29	Female	None found, but strong family history	None	Prelingual	Cochlear CI612

*Absent corpus collosum and undiagnosed syndromic facial features. ^†^Prematurity of 28 weeks, twin-to-twin transfusion syndrome, special care unit admission for 10 weeks, phototherapy for jaundice, and acquired cytomegalovirus. ANSD = auditory neuropathy spectrum disorder; NICU = neonatal intensive care unit

Patients were reviewed via both video link and face to face, as per our protocol. None of the children or parents developed Covid-19 symptoms in the period between the surgery and switch-on of the implant (three weeks), and no complications related to Covid-19 were recorded.

Virtual follow-up duration ranged from 7 to 10 days post-operatively, with switch-on 3 weeks post-operatively. For patients who underwent more than one operation, the follow-up interval was calculated from the date of the most recent procedure.

The early age group included four children with an age at implantation ranging from 8 to 15 months. The second group included seven patients with an age range from 19 to 37 months at implantation.

There were variations between the children included in this study in terms of the length of time before implantation. The delays in the study cohort were because of patients being unwell, the need for interventions or investigations such as ABR and grommets, and an already high failure to attend rate before the coronavirus disease crisis.

## Discussion

The continuation of cochlear implantation in children during the Covid-19 pandemic is essential. It is dependent on the availability of staff, PPE and other safety precautions. Demonstrating such feasibility is relevant both nationally and internationally, given the unpredictable nature of the Covid-19 pandemic and the possibility of a resurgence of cases in the medium- to long-term.^3^ The impact that Covid-19 has had on delays in audiological assessment is important to factor in when considering the overall pathway from referral to implantation.

Within the UK, there has been regional variation in implantation during the ongoing Covid-19 pandemic (Prof Helen Cullington, British Cochlear Implant Group Chair, personal communication with permission, 22nd June 2020). New cases of Covid-19 have emerged at differing times and intensive care unit occupation has varied geographically.^29^ Hospitals in large conurbations such as London appear to have been the most overwhelmed. Where capacity allows, it is important to prioritise surgical operations according to NHS guidance. This guidance divides procedures into four categories depending on the urgency, ranging from those procedures that need to be performed within 24 hours (category 1a) to those that could be delayed for more than 3 months (such as tympanoplasty) (category 4).^12^

Delays can take place at any stage of the pathway (even before the Covid-19 crisis). There can be delays in referrals, and delays due to: a failure of children to attend, the need for repeat testing and parental anxiety. Delays in this study cohort occurred even though no children who were referred for assessment failed to attend.

All these factors exert additional pressures on cochlear implant programmes to successfully complete implantation within a predetermined timeframe. These factors are reflected in the time taken from referral to surgery. The time from the MDT's decision to operate to the time of surgery reflects other factors such as operating theatre list availability, surgical availability and case prioritisation. This time was shorter in our hospital during the Covid-19 crisis mainly because of the cancellation of all routine operating theatre activities, including other otological procedures.

There is the potential risk of transmission or development of Covid-19 during the peri-operative period, but the precise risk is still unknown. Three hundred category 3 surgical procedures (which are to be carried out within three months^12^) were performed during this period in our hospital. Of these patients, three were subsequently re-admitted to hospital and found to be Covid-19 positive. There were no intensive treatment unit admissions or mortalities associated with this cohort. The hospital has, as an additional precaution, introduced a 14-day period of pre-operative self-isolation.

During the shared decision-making process of consent, these risks should be clearly communicated to parents and balanced against the lifelong benefit of early implantation. The uncertainty regarding surgical related Covid-19 morbidity and mortality needs to be discussed with the parents (and documented).^30^ Parents of children included in this study had understandable concerns about the risks of surgery during the pandemic. They were counselled with best available evidence and they were all keen to proceed once the long-term negative effects of delaying the procedure were discussed with them.

It is important to consider the effects of Covid-19 on service delivery; namely, the uncertainty about the timing of service recommencement and the workload that will be created as a result of the cancellation of routine services. All these factors necessitate careful planning and prioritisation, both during the crisis and afterwards. There is particular concern in the media that the impact on screening for cancer and the early diagnosis of cancer will lead to a higher mortality rate caused by delays and more advanced disease. Cochlear implantation in children is equally time-sensitive in terms of obtaining the best outcomes, and the service must be maintained. The time sensitivity of cochlear implantation in prelingually deafened children should be reflected in future cochlear implant guidelines. Experience gained from operating on children who need cochlear implantation may also facilitate NHS trusts introducing other procedures once routine services recommence.

Some children's surgery could be delayed further because of the cancellation of investigations in the referring hospital (such as ABR under general anaesthesia). Hence, it may be advisable to arrange for such investigations in the cochlear implant centre itself, to avoid such unnecessary delays. To complicate matters further, in some hospitals (including our centre), audiology and out-patient teams may be re-deployed elsewhere. It is important to highlight the important roles of these teams in ensuring a successful cochlear implantation (including intra-operative assessment and post-operative reviews). In complicated cases, the patient pathway from diagnosis to cochlear implantation can be a lengthy one, and further delays because of Covid-19 could have a detrimental long-term result, as demonstrated in the literature.^16,17^

Length of hospital stay is important and could have long-term implications after the reintroduction of the service. Analysis of pathways and changes, such as the introduction of same-day discharge (with same-day X-ray confirmation of implant placement) followed by remote video review the next day from home, should be considered.

Our centre covers a large geographical region and includes some of the most socioeconomically deprived areas in the country.^31^ As a result, there are considerable delays in diagnosis, delays in referrals, and a high failure to attend rate of children who are referred. In addition to all this, during the Covid-19 period, we have had additional problems with non-attendance in local paediatric audiology departments given their limited capacity and parental anxiety about attendance. Our experience, however, showed that by tailoring management in this limited number of patients, by telephoning parents and explaining the need and the time-critical nature of their child's surgery, we succeeded in achieving a 0 per cent failure to attend rate.

Future delays are almost inevitable, especially in programmes that stopped implanting during lockdown (Prof Helen Cullington, British Cochlear Implant Group Chair, personal communication with permission, 22nd June, 2020), and are only starting to attempt to clear their backlog because of the recent easing of restrictions in England. However, there is a possibility of additional peaks,^3^ with further shutdowns, before these newly diagnosed patients have been implanted. If programmes shut down again, these children may well be pushed beyond the time-critical period for implantation. There is also variation in the timing of the disease peaks across the world.^2^

It is important to report our preliminary findings showing that cochlear implantation surgery in children is feasible, despite the additional burden that the Covid-19 pandemic has placed on audiological resources and operating theatre capacity. The continued success of an implant programme in times of crisis, when the reallocation of resources occurs, is largely dependent on the support of departmental colleagues and the strategic foresight of individual hospital management.

### Limitations

Post-operative Covid-19 swabs were not performed. However, follow up of all our patients showed that none symptomatically developed Covid-19. Patient-reported outcome measures were not used in this study; their use in future studies is recommended. The study does not include procedures for implanting Covid-19 positive children, and none of the children included in this study tested positive. Patients scheduled to undergo other non-emergency surgical procedures in our centre who tested positive for Covid-19 had their operations postponed until a negative test result was obtained.

• The long-term outcomes of cochlear implantation in prelingually deafened children are worse if surgery is delayed
• The current coronavirus disease 2019 pandemic has significantly restricted the provision of elective surgical practice
• As with cancer management, cochlear implantation in children is time-sensitive and must be prioritised
• Essential multidisciplinary team-working, required for successful surgery and rehabilitation, is challenging if patients and staff are to be kept safe from infection
• Our methodology for maintaining the service during the pandemic onset is presented

Our study is relatively small, but it provides a basis for further ongoing work in this area. It describes a solely UK experience, but carries an important international message.

## Conclusion

Implantation during the Covid-19 pandemic in the paediatric population is feasible. Careful planning for all stages of the patient pathway is necessary to minimise risk through exposure, and telemedicine is a useful adjunct in this regard. The time sensitivity of cochlear implantation in prelingually deafened children should be reflected in future cochlear implant guidelines.
